# Mitochondrial homeostasis: a potential target for delaying renal aging

**DOI:** 10.3389/fphar.2023.1191517

**Published:** 2023-06-15

**Authors:** Ming Yang, Chongbin Liu, Na Jiang, Yan Liu, Shilu Luo, Chenrui Li, Hao Zhao, Yachun Han, Wei Chen, Li Li, Li Xiao, Lin Sun

**Affiliations:** ^1^ Department of Nephrology, The Second Xiangya Hospital of Central South University, Changsha, China; ^2^ Hunan Key Laboratory of Kidney Disease and Blood Purification, Changsha, Hunan, China

**Keywords:** renal aging, mitochondria, mitophagy, oxidative stress, inflammation

## Abstract

Mitochondria, which are the energy factories of the cell, participate in many life activities, and the kidney is a high metabolic organ that contains abundant mitochondria. Renal aging is a degenerative process associated with the accumulation of harmful processes. Increasing attention has been given to the role of abnormal mitochondrial homeostasis in renal aging. However, the role of mitochondrial homeostasis in renal aging has not been reviewed in detail. Here, we summarize the current biochemical markers associated with aging and review the changes in renal structure and function during aging. Moreover, we also review in detail the role of mitochondrial homeostasis abnormalities, including mitochondrial function, mitophagy and mitochondria-mediated oxidative stress and inflammation, in renal aging. Finally, we describe some of the current antiaging compounds that target mitochondria and note that maintaining mitochondrial homeostasis is a potential strategy against renal aging.

## 1 Introduction

With the development of the social economy and improvements in modern medical technology, human life expectancy has been greatly increased, but the accompanying problem is that the level of social aging increased. Globally, there are an estimated 901 million people aged 60 years or older, and this number is expected to rise to 2 billion by 2050 (Bridges and Zalups, 2017). The aging of society has led to an increase in the prevalence of multiple chronic diseases, including cardiovascular diseases (Paneni et al., 2017), neurodegenerative disease (Gonzales et al., 2022), cancers (Campisi, 2013) and metabolic diseases (Franceschi et al., 2018; Wang et al., 2022a). Generally, aging can be divided into two categories: physical aging and pathological aging. The former refers to the process of physical degeneration, while the latter refers to the senile changes caused by various factors such as diseases. Cell senescence was originally defined as the loss of replication potential in primary cells during culture and the development of DNA damage due to telomere dysfunction (Miwa et al., 2022). Aging cells are eliminated by the immune system, and new cells are constantly created from the appropriate tissues and organs to make up for aging cells. The dynamic balance between cell senescence, death and new cell growth is the basis of maintaining the normal life activities of organisms. Although not identical, cellular senescence is the basis of physical aging, so the goal of antiaging is to delay or reverse cellular senescence. The kidney is one of the most metabolically active organs, and aging can lead to a series of changes in renal function reducing in the kidney’s ability to respond to external challenges (Meyer, 1989; Noordmans et al., 2015; Schmitt and Melk, 2017). Therefore, a deeper understanding of the molecular mechanism of kidney aging is beneficial for delaying kidney aging and preventing renal injury.

Mitochondria are the oldest organelles in eukaryotes. It is a bilayer membrane structure, including the outer mitochondrial membrane (OMM) and inner mitochondrial membrane (IMM). The OMM contains a variety of proteins and pores that mediate the exchange of signal materials between mitochondria and cytoplasm. The IMM invaginates multiple folds to form mitochondrial cristae and contains a large number of enzymes. This enlarges the inner membrane of the mitochondria and allows it to function better. Mitochondria are involved in many processes, including fatty acid oxidation, ATP synthesis, calcium ion transport, and steroid synthesis (Qu et al., 2016; Bhatti et al., 2017; Roger et al., 2017). Normal mitochondrial structure and function are the basis of maintaining cell homeostasis. Mitochondrial dysfunction is also thought to be involved in the progression of aging, and the mitochondrial free radical theory of aging (MFRTA) has taken center stage for decades in aging theory (Sanz and Stefanatos, 2008; Sanz et al., 2010; Son and Lee, 2019). This theory holds that mitochondria produce a large number of reactive oxygen species during oxidative phosphorylation, and the increased reactive oxygen species can lead to oxidative damage in a variety of proteins, lipids and nucleic acids. Moreover, damaged mitochondrial DNA (mtDNA) will further aggravate the oxidative abnormality, forming a vicious cycle. This theory is based on the fact that the level of intracellular reactive oxygen species increases with age; the activity of ROS scavenging enzymes decreases with increasing age; mtDNA mutations increase with aging; long-lived animals produce fewer free radicals and have lower levels of oxidative tissue damage. The kidney is an organ with abundant mitochondrial levels. A disturbance in mitochondrial homeostasis in the kidney can accelerate the aging of the kidney, while maintaining mitochondrial homeostasis can effectively delay the aging process in the kidney (Miao et al., 2019). Therefore, we review the importance of mitochondria in delaying renal aging to target mitochondria as an effective strategy for delaying of renal aging.

## 2 Markers of cellular senescence

In aging research, *in vivo* and *in vitro* biomarkers are essential. However, due to the lack of an in-depth understanding of aging, there is still a lack of a general and specific detection indices. Currently, multiple treatments are is commonly used to determine whether aging occurs.

### 2.1 p16^INK4a^

p16^INK4a^ is a potent inhibitor of the G1 phase transition of the cell cycle. It directly binds cyclin-dependent kinase 4/6, inhibits its kinase activity, prevents phosphorylation of the retinoblastoma tumor suppressor, and further inhibits E2F-mediated transcription leading to cell cycle arrest (D'Arcangelo et al., 2017). In mammals, the expression of p16^INK4a^ is highly dynamic, and its expression increases exponentially with age (Zindy et al., 1997; Krishnamurthy et al., 2004; Melk et al., 2004), while inhibiting the expression of p16^INK4a^ can effectively delay the aging phenotype (Baker et al., 2011).

### 2.2 γH2AX

DNA damage is one of the mechanisms that induces cell aging. In aging or other diseases, due to a large number of exogenous and endogenous gene toxins, DNA damage will continue to occur on a large scale, and the damaged DNA will further aggravate the occurrence of aging (Schumacher et al., 2021; Yousefzadeh et al., 2021). Double-strand breaks (DSBs) occur in a cell when two strands of DNA are close enough together (<20 bp). This occurrence destroys the linear continuity of the genome, which is one of the critical lesions related to cell survival and genome integrity preservation (Mah et al., 2010). After that happens, cells can repair the damage through the DNA damage response (DDR), which mainly involves, homologous recombination (HR) and nonhomologous end joining (NHEJ), to avoid the adverse effects of DSBs (Chang et al., 2017; Wright et al., 2018; Polleys and Freudenreich, 2021). The cell’s early response to DSBs is the phosphorylation of H2AX, a variant of group protein H2A, whose C-terminal Ser-139 is phosphorylated to produce γH2AX (Sharma et al., 2012; Ibuki and Toyooka, 2015). This factor can participate in the DSB repair process by increasing DNA accessibility and promoting the recruitment and accumulation of specific DDR proteins at the end of the DNA. Telomere length is shortened during aging, which requires timely repair via γH2AX. Therefore, γH2AX expression is gradually increased during aging, and related results have been observed *in vivo* and *in vitro* (Merz et al., 2019; Cai et al., 2021). However, in addition to aging, γH2AX expression is increased in some pathological conditions, so it still has certain limitations as a specific indicator of aging.

### 2.3 Senescence-associated beta-galactosidase (SA-β-gal) assay

Aging cells express β-galactosidase activity, which can be detected by histochemistry at a pH of 6.0 (Itahana et al., 2007). Currently, this has become the most common method to detect senescent cells, but its disadvantage is that it can only detect the senescence levels of fresh or frozen tissue, rather than formalin-fixed paraffin-embedded archival tissue samples.

### 2.4 Lipofuscin

Lipofuscin is a complex of highly oxidized cross-linked macromolecules (proteins and lipids) with multiple metabolic sources in the cell (Moreno-Garcia et al., 2018). It cannot be degraded or cleared by exocytosis, and so it accumulates inside the cell. In cells with active proliferation, lipofuscin can be effectively diluted by cell division, but shows little pigment accumulation, while in aging cells, continued accumulation leads to pigmentation, especially in nerve cells, heart muscle cells, and skin cells (Brunk and Terman, 2002; Porta, 2002; Firlag et al., 2013). Kakimoto et al. collected 76 heart samples from autopsy patients aged 20–97 years and found that lipofuscin accumulated primarily in the perinuclear region and that the accumulation rate was positively correlated with aging (Kakimoto et al., 2019).

In addition, p53 and p21 expression levels are often used to assess the level of aging. p53 is a major player in the DDR pathway and a key regulator of the cell cycle that activates cyclin dependent kinase inhibitors (CDKIs) and ultimately leads to cell cycle arrest (Schmid et al., 2006). p21 is a downstream molecule of phosphorylated p53 that can block cell cycle entry into G1/S phase to act as a cell cycle inhibitor (Hernandez-Segura et al., 2018). Moreover, changes in Ki-67 and lamin B1 levels are also commonly used to assess aging phenotypes (Krupp et al., 2000; Matias et al., 2022).

## 3 Changes in renal structure and function in aging

In the aging state, renal structure including glomeruli, renal tubules and renal blood vessels, are changed to varying degrees. Gourtsoyiannis et al. used CT wo show that the thickness of the kidney parenchyma decreased by approximately 10% for each additional 10 years of age in men and women without kidney disease (Gourtsoyiannis et al., 1990). A similar result showed that the renal size decreased with age, which was almost entirely due to the reduction in the renal parenchyma, as shown by ultrasound (Emamian et al., 1993). Among microanatomical changes, the kidneys show a decrease in the number of nonsclerosing glomeruli, a decrease in the number of renal tubules, and changes in blood vessels in healthy aging individuals (Darmady et al., 1973). In living donor kidneys, the prevalence of globally sclerotic glomeruli (GSGs) increases with age, with a GSG prevalence of 19% in kidney donors aged 18–29 years and 82% in donors aged 70–77 years (Denic et al., 2016). As the number of sclerosed glomeruli increases, the remaining glomeruli develop compensatory hypertrophy to maintain normal renal function, and functional nephrons decrease with age (Denic et al., 2016). In addition to morphological changes, the function of aging kidneys also changes. The estimated glomerular filtration rate (eGFR), which is often used to estimate kidney function, begins to show an age-induced decline after age 30 and declines at a rate of about approximately 1 mL per year. A study of 610 people over 70 years of age showed that half had an eGFR of less than 60 mL/min/1.73 m^2^ (Schaeffner et al., 2012).

In general, a large number of nephrons will atrophy and lose function, which leads to a decline in the ability of the kidney to resist external stimuli in aging kidneys. In addition, the elderly suffer from a variety of other diseases, which lead to an increase in drug doses, and renal toxicity and the excretion of some drugs further increase the burden on the kidney. Therefore, the renal function of elderly patients should be given special attention in clinical practice. NSAIDs and contrast agents should be used cautiously to avoid renal injury. Aging weakens the ability of the kidney to resist external stimuli, so it is more vulnerable to damage. Therefore, in-depth understanding of the mechanism of kidney aging and effectively delaying of kidney aging is a strategy to reduce the incidence of kidney diseases.

## 4 Mitochondria and renal aging

The kidney, which is an organ with extremely vigorous metabolism, contains abundant mitochondria to provide a large amount of energy to maintain the functions of renal excretion and reabsorption (Yang et al., 2021a). When the kidney is stimulated, the mitochondria produce more ATP in response to adverse stimuli. In addition, damaged mitochondria can be promptly cleared through mitophagy. In the aging state, when mitochondrial ATP synthesis ability and mitophagy are impaired, damaged mitochondria release a large amount of mitochondrial contents, leading to cellular oxidative stress and other adverse effects (Yang et al., 2021a). Here, we will focus on the role of mitochondrial structure and function disorders in renal aging.

### 4.1 Mitophagy in renal aging

Mitophagy is a process in which cells selectively degrade excessive or damaged mitochondria through the lysosome pathway (Ashrafi and Schwarz, 2013; Kerr et al., 2017; Lou et al., 2020). In mammals, the most well-studied mitophagy pathway is the PINK1/Parkin pathway. When mitochondrial damage results in decreased membrane potential, PINK1 accumulates in the mitochondrial outer membrane and cannot be degraded. PINK1 is phosphorylated to recruit Parkin for translocation to the mitochondrial outer membrane. Parkin-mediated ubiquitination of a variety of mitochondrial proteins causes them to associate with the ubiquitin-binding domain of autophagy receptors and form autophagosomes, ultimately mediating mitochondrial degradation by lysosomes (Eiyama and Okamoto, 2015; Quinn et al., 2020). Moreover, PINK1 can also directly recruit autophagy receptors, thereby mediating mitophagy (Lazarou et al., 2015). In addition to mitophagy mediated by PINK1/Parkin, other mitophagy proteins, such as activating molecule in beclin1-regulated autophagy (AMBRA1) (Gambarotto et al., 2022), BCL2-interacting protein 3 (BNIP3) (Vara-Perez et al., 2021), FUN14 domain containing protein 1 (FUNDC1) (Pei et al., 2021) and NIP3-like protein X (NIX) (Wu et al., 2021), mediate the occurrence of mitophagy in different states.

The continuous accumulation of dysfunctional mitochondria is an important sign of aging, and as a means of removing damaged mitochondria, abnormal mitophagy plays an indispensable role in the aging process. Studies have shown that the levels of mitophagy in the dentate gyrus, heart, and skeletal muscle were significantly reduced in older mice compared to controls (Hoshino et al., 2013; Sun et al., 2015; Garcia-Prat et al., 2016), and similar results were observed in the heart and skeletal muscle of older humans (Martinez-Lopez et al., 2015; Shirakabe et al., 2016; Guo and Chiang, 2022). Moreover, the RNA-binding protein Pumilio2 (PUM2) is a negative regulator of longevity and healthy lifespan that is induced during aging by inhibiting the mRNA expression of mitochondrial fission factor (MFF), thereby impacting mitochondrial fission and mitophagy. PUM2 knockout enhances mitochondrial fission and autophagy levels in mice, thereby improving mitochondrial homeostasis and delaying senescence (D'Amico et al., 2019). The enhancement of mitophagy can effectively delay the aging process. In *C. elegans*, the accumulation of dysfunctional mitochondria occurs with aging and longevity, and treatment with UA (a natural mitophagy agonist) can effectively ameliorate these adverse changes and delay aging (Ryu et al., 2016). Similarly, Pyo et al. found that overexpression of ATG5, an autophagy protein, enhanced autophagy levels and significantly extended mouse lifespans. In addition, mouse embryonic fibroblasts isolated from ATG5 transgenic mice showed increased oxidative stress resistance, which was reversed by autophagy inhibitors (Pyo et al., 2013). These results indicate that the level of mitophagy in aging tissues is decreased, and enhancing mitophagy to remove damaged mitochondria in time is a key means to delay aging.

The kidney is an organ with abundant mitochondria, and the relationship between abnormal mitophagy and kidney aging has been fully revealed. Mitophagy, especially that mediated by the classical PINK1/Parkin pathway, is conducive to the timely removal of dysfunctional mitochondria, thus slowing kidney injury and the aging process. Abnormal mitophagy in various renal diseases aggravates the aging of renal cells. In diabetic nephropathy or acute kidney injury (AKI), the expression of the aging markers p16 and SA-β-gal in renal tubule cells was increased, which was accompanied by a decrease in PINK1, Parkin and LC3 expression levels (Chen et al., 2018; Bae et al., 2020). Wang et al. showed that the expression of legumain (a conserved lysosomal peptidase) in the kidney is significantly downregulated with age (Wang et al., 2022b). In renal tubulo-specific legumain-knockout mice or legumain-knockout HK-2 cells, this deficiency induces tubular cell senescence, which increases the secretion of fibrosis-related cytokines and ultimately accelerates fibroblast activation (Wang et al., 2022b). Mechanistically, downregulating legumain could enhance lysosome function, thus providing timely clearing of damaged mitochondria. This conclusion is supported by the fact that lysosome function is abnormal and mitophagy is impaired in the tubule cells of aged legumain-knockdown mice (Wang et al., 2022b). A similar result was observed in the DN state, which treatment of Mdivi-1 (an inhibitor of mitochondrial fission/mitophagy) enhanced the senescence of renal tubular epithelial cells (RTECs) under HG conditions, whereas Torin1 (a mitophagy agonist) notably inhibited RTEC senescence (Chen et al., 2018). Moreover, the downregulation of optineurin (OPTN) significantly inhibited mitophagy under HG conditions, while overexpression of OPTN alleviated cell aging by promoting mitophagy (Chen et al., 2018). Interestingly, the expression of OPTN was negatively correlated with the renal tubule interstitial injury score in clinical specimens, and the aging marker protein p16 was not expressed in OPTN-positive renal tubule cells (Chen et al., 2018). In addition, quercetin could relieve angiotensin II (AngII) -induced RTEC senescence by activating Sirt1/PINK1/Parkin-mediated mitophagy (Liu et al., 2020). Recently, dietary restriction has been shown to ameliorate renal aging, possibly by enhancing mitophagy. Cui et al. demonstrated that the expression of mitophagy marker proteins was significantly decreased in the kidneys of mice in the high-calorie diet group, which was accompanied by severe morphological abnormalities in mitochondria and increased markers of aging, whereas caloric restriction could enhance mitophagy levels (upregulated PINK1, Parkin and LC3 expression) and attenuate oxidative damage and aging in the kidney (Cui et al., 2013). A similar result was showed that calorie restriction could enhance mitophagy to delay renal aging in rat models (Andrianova et al., 2018). This evidence reveals the importance of maintaining mitophagy in renal aging, and maintaining mitophagy activity is conducive to the timely removal of damaged mitochondria, thus preventing further mitochondrial dysfunction, mitochondria-mediated inflammation and renal injury. Thus, effective mitophagy activators may be agents to delay the progression of renal aging.

### 4.2 Mitochondria-mediated inflammation in renal aging

Inflammation is the immune system’s response to the invasion of viruses, bacteria and other pathogens. However, when the immune system is not properly regulated, it can also cause damage to the body itself (Medzhitov, 2008; Feehan and Gilroy, 2019). Excessive inflammation is present in a variety of age-related chronic diseases, including Alzheimer’s disease (Akiyama et al., 2000), atherosclerosis (Wolf and Ley, 2019) and diabetes (Rohm et al., 2022). With further research on the mechanism of aging, the occurrence of inflammation is believed to accelerate the progression of aging. Elderly individuals often exhibit a state of high inflammation, which is characterized by elevated levels of circulating proinflammatory cytokines, and several epidemiological studies have shown strong associations between inflammation and some of the pathology associated with aging (Licastro et al., 2005; Singh and Newman, 2011; Tran et al., 2016). Therefore, anti-inflammatory therapy is considered to be an important strategy for delaying aging.

In cells, damaged mitochondria activate inflammation through different signaling pathways. In response to external stimulation, the leakage of large amounts of mitochondrial contents can directly activate inflammatory responses. Unlike the nuclear DNA, mtDNA (and bacterial DNA) is not methylated, and the immune system has evolved to recognize unmethylated DNA and quickly become activate (Yan et al., 2019; Pereira et al., 2021). On the one hand, mtDNA activates the NLRP3 inflammasome, which in turn facilitates IL-1β- and IL-18-mediated inflammation (Yang et al., 2021b; Luo et al., 2022). Free mtDNA in the cytoplasm is recognized by the cytoplasmic sensor cGAS and activates the transcription factor interferon regulatory factor 3 (IRF3) via stimulator of interferon genes (STING) and TANK binding kinase 1 (TKB1), inducing the production of type 1 interferon (IFN) and IFN-stimulated gene products (Sun et al., 2016). In addition to mtDNA, mitochondria are the sites of intracellular metabolism, producing large amounts of ROS during oxidative metabolism, which is an important cause of intracellular inflammation (Sinha et al., 2013; Yang et al., 2016; Cadenas, 2018). In addition, mitochondria activate innate immune responses. The outer mitochondrial membrane contains mitochondrial antiviral signaling protein (MAVS), which mediates the recognition of viral RNA and triggers the signal transduction cascade that drives the production of type I interferons (Green et al., 2011). Therefore, mitochondria play an essential role in cellular and inflammatory responses.

The relationship between inflammation and kidney aging is attracting increasing attention. There was increased renal fibrosis, inflammation and cell senescence in unilateral ureteral obstruction (UUO) and unilateral ischemia‒reperfusion injury (UIRI) rat models, and these adverse changes could be reversed by denervation (Li et al., 2021). Mechanistically, norepinephrine could induce epithelial cells to secrete proinflammatory cytokines and accelerate cell aging by activating α2A-adrenergic receptor (α2A-AR) (Li et al., 2021). Similarly, Bai et al. demonstrated that miR-335 and miR-34a could inhibit the expression of superoxide dismutase 2 (SOD2) and thioredoxin reductase 2 (Txnrd2) by binding to the 3' -untranslated region of the gene, leading to an increase in mitochondrial reactive oxygen species, increased inflammation, and premature aging in renal mesangial cells (Bai et al., 2011). Moreover, compared with those in young rats, ROS levels and inflammation were increased in the kidneys of elderly rats, while MHY3200 (a PPARα agonist) intervention could effectively activate PPARα and inhibit NF-κB, thus inhibiting the occurrence of kidney inflammation (Kim et al., 2021). Moreover, Kim et al. showed that resveratrol treatment activated the Nrf2 and sirt1 signaling pathways in the kidneys of aging mice, thereby alleviating renal oxidative stress, mitochondrial dysfunction and inflammation (Kim et al., 2018). Unfortunately, although the role of mtDNA in aging has been demonstrated in other tissues (Pinto and Moraes, 2015; Quan et al., 2020), its association with aging has not been reported in the kidney.

### 4.3 Mitochondrial function in renal aging

**FIGURE 1:**
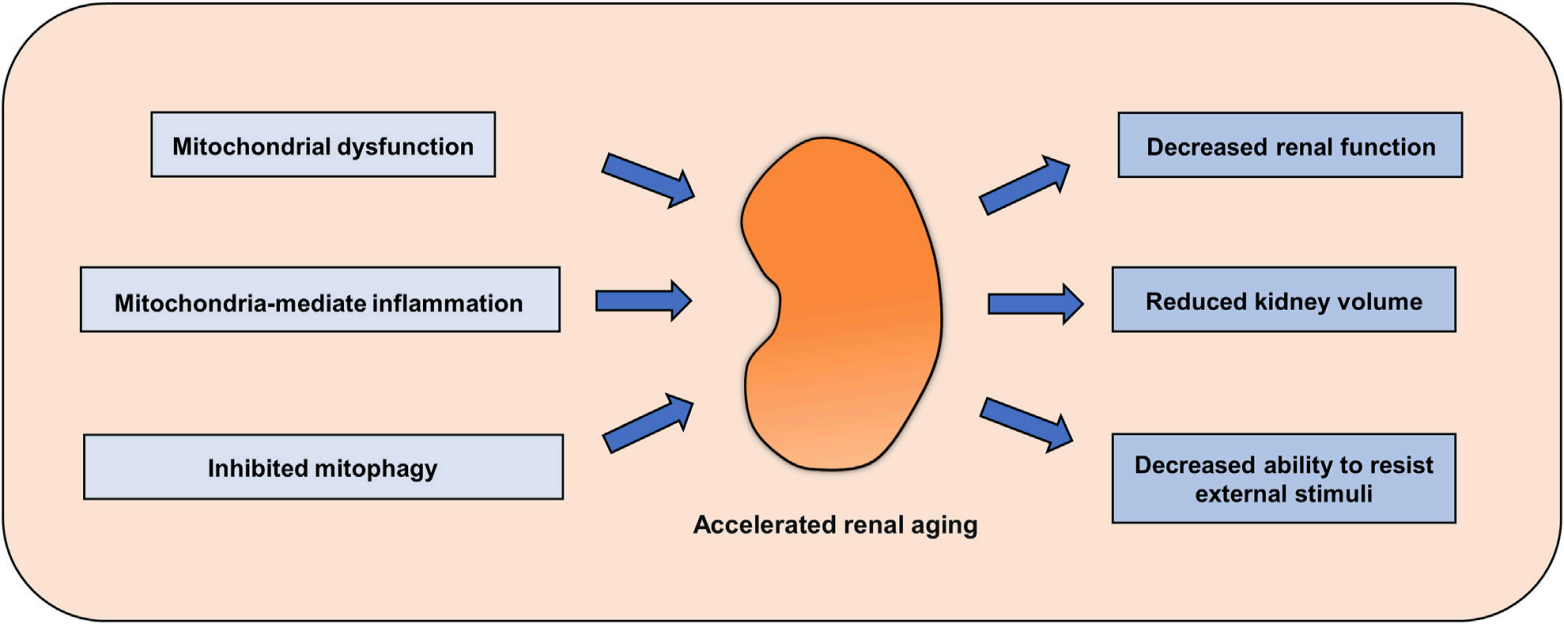
Renal aging mediated by abnormal mitochondrial homeostasis. Abnormal mitochondrial function, decreased mitophagy and enhanced mitochondria-mediated inflammation accelerate renal aging, decreasing kidney function and the ability to resist adverse external stimuli.

Mitochondria are the energy factories of cells, and so the most important function of mitochondria is to generate a large amount of ATP through the oxidative phosphorylation pathway for cells to carry out various activities. With increasing age, mitochondrial function gradually declines, which is followed by a decline in cell activity. Mitochondrial dysfunction often occurs before aging, and when the function of a certain number of mitochondria in a cell is disturbed, cell aging occurs. Mitochondria may produce a large amount of ATP through oxidative phosphorylation to resist the stimulation of some aging factors and thus delay the aging process. Therefore, enhancing mitochondrial function may be a strategy to delay cell senescence. There was impaired mitochondrial function in the renal cortex, disturbed redox homeostasis and decreased renal function in the kidney of aged rats compared to control (Mohammad et al., 2022). Moreover, overexpression of Klotho could restore mitochondrial functional homeostasis, inhibit ROS production and enhance the expression of mitochondrial respiratory chain complex subunits, thus improving the occurrence of renal aging and fibrosis (Miao et al., 2021). A similar result was showed that increasing mitochondrial biogenesis and mitochondrial function could significantly alleviate the renal aging phenotype (Miao et al., 2019; Zhu et al., 2022). The expression of cannabinoid receptor 2 (CB2), which is responsible for activation of the endocannabinoid system, is increased in the kidney during aging and is accompanied by a decline in mitochondrial mass. Deletion of the CB2 gene in aging mice significantly inhibits the activation of β-catenin signaling and restores mitochondrial function and adenosine triphosphate production, thereby ameliorating the renal aging phenotype (Zhou et al., 2021). In addition, a healthy lifestyle is a strategy to improve mitochondrial function and delay aging. Physical exercise is a well-established way to improve metabolism and mitochondrial function. Several studies have shown that exercise can effectively improve mitochondrial function and delay aging of the heart, muscle, bone and kidney (Garatachea et al., 2015; Hood et al., 2019; Memme et al., 2021). Moreover, intermittent fasting, another popular lifestyle option, can boost mitochondrial function to slow the onset of aging (de Cabo and Mattson, 2019; Zhao et al., 2022). These results indicate that enhancing mitochondrial function can effectively delay the process of renal aging (Figure 1). At present, with the in-depth study of mitochondria, a variety of mitochondrial protective agents have been identified, and their role in delaying aging has been partially revealed, which is summarized in the next section.

## 5 Antiaging compounds that target mitochondria

With increasing attention being paid to the role of mitochondrial homeostasis in aging, antiaging drugs targeting mitochondria have been developed. Metformin is a clinically used hypoglycemic drug, and its effects beyond hypoglycemia have attracted attention, studies have shown that it can also protect mitochondrial function. Metformin can upregulate the level of mitophagy, improve mitochondrial energy metabolism and ameliorate cellular inflammation (Bharath et al., 2020; Chowdhury et al., 2020). In addition, Montalvo et al. showed that metformin increased the antioxidant capacity of mitochondria and reduced the accumulation of oxidative damage and chronic inflammation, thus delaying aging (Martin-Montalvo et al., 2013). In the kidney, a similar result was observed: metformin slowed the aging of renal tubular epithelial cells in diabetic nephropathy by activating the MBNL1/miR-130a-3p/STAT3 pathway (Jiang et al., 2020) In addition to metformin, a variety of mitochondrial protectors have been shown to slow the aging process. Here we summarized some compounds (Table 1).

**TABLE 1:** Antiaging compounds that target mitochondria.

Number	Name	Effects	Ref
1	Metformin	Mitochondrial function and mitophagy	Podhorecka et al. (2017), Mohammed et al. (2021)
2	Nicotinamide mononucleotide (NMN)	Mitochondrial function and inflammation	Nadeeshani et al. (2022)
3	Resveratrol	Mitochondrial oxidative stress	Kim et al. (2018)
4	Urolithin A	Autophagy	Chen et al. (2019a)
5	Lycopene	Mitochondrial oxidative stress	Liu et al. (2018)
6	Alpha-ketoglutarate	Tricarboxylic acid cycle	Wang et al. (2020)
7	N-acetylcysteine (NAC)	Oxidative stress	Zhu et al. (2021)
8	Aronia melanocarpa	Inflammation	Zhao et al. (2021)
9	S-Allylcysteine	Mitochondrial function	Chen et al. (2022)
10	Spermidine	Mitochondrial function	Jing et al. (2018)
11	Leonurine	Oxidative stress	Chen et al. (2019b)
12	T1-11	Inflammation	Hsu et al. (2020)
13	Ursolic Acid	Mitochondrial function	Bahrami and Bakhtiari (2016)
14	ProBeptigen/CMI-168 (PB)	Inflammation	Ni et al. (2021)
15	Astaxanthin	Oxidative stress	Wu et al. (2014)
16	Taurisolo	Oxidative stress	Annunziata et al. (2020)

## 6 Conclusion

Aging is an inevitable life process. The ability of aging organs to resist adverse external stimuli decreases, and thus, they are more vulnerable to damage. Mitochondrial homeostasis plays an indispensable role in maintaining kidney function, and when mitochondrial function is disturbed, it will accelerate the aging of renal cells. Here, we reviewed the evidence of renal mitochondrial disorders, including abnormal mitochondrial function, abnormal mitophagy, and abnormal activation of oxidative stress and inflammation in renal aging. Although targeting mitochondria is a potential strategy to slow kidney aging, many questions remain to be addressed, such as what role mtDNA plays in renal aging. Although current studies have shown that inhibiting the release of mtDNA can inhibit the activation of inflammation and the occurrence of aging in other tissues and cells, there has been no research on the role of mtDNA in renal aging. Moreover, identifying specific renal mitophagy activators is also needed. Current studies have been conducted on animal and cell models, and there may be significant differences in the aging process and immune system between species that could limit the applicability of these findings to humans. Therefore, the relationship between aging and mitochondrial abnormalities in kidney tissue needs to be clarified in future studies. When these questions are thoroughly investigated and answered, targeting mitochondria as a strategy to alleviate kidney aging will be viable. In addition, given the importance of mitochondria in biological activity and aging, targeting mitochondria may be a strategy for delaying aging in organs other than the kidney.
